# Herbal compound "Songyou Yin" reinforced the ability of interferon-alfa to inhibit the enhanced metastatic potential induced by palliative resection of hepatocellular carcinoma in nude mice

**DOI:** 10.1186/1471-2407-10-580

**Published:** 2010-10-25

**Authors:** Xiu-Yan Huang, Zi-Li Huang, Lu Wang, Yong-Hua Xu, Xin-Yu Huang, Kai-Xing Ai, Qi Zheng, Zhao-You Tang

**Affiliations:** 1 Department of General Surgery, 6th People's Hospital of Shanghai, Shanghai Jiaotong University, Shanghai 200233, PR China; 2 Department of Radiology, Central Hospital of Shanghai Xuhui District, Shanghai 200031, PR China; 3 Liver Cancer Institute and Zhongshan Hospital, Fudan University, Shanghai 200032, PR China; Key Laboratory for Carcinogenesis & Cancer Invasion, the Chinese Ministry of Education, Shanghai 200032, PR China

## Abstract

**Background:**

Liver resection is a widely accepted treatment for hepatocellular carcinoma (HCC). Our previous clinical study showed that the rate of palliative resection was 34.0% (1958-2008, 2754 of 8107). However, the influence of palliative resection on tumor metastasis remains controversial. The present study was conducted to evaluate the effect of palliative resection on residual HCC and to explore interventional approaches.

**Methods:**

Palliative resection was done in an orthotopic nude mice model of HCC (MHCC97H) with high metastatic potential. Tumor growth, invasion, metastasis, lifespan, and some molecular alterations were examined in vivo and in vitro. Mice that underwent palliative resection were treated with the Chinese herbal compound "Songyou Yin," interferon-alfa-1b (IFN-α), or their combination to assess their effects.

**Results:**

In the palliative resection group, the number of lung metastatic nodules increased markedly as compared to the sham operation group (14.3 ± 4.7 versus 8.7 ± 3.6, *P *< 0.05); tumor matrix metalloproteinase 2 (MMP2) activity was elevated by 1.4-fold, with up-regulation of vascular endothelial growth factor (VEGF) and down-regulation of tissue inhibitor of metalloproteinase 2 (TIMP2). The sera of mice undergoing palliative resection significantly enhanced cell invasiveness by 1.3-fold. After treatment, tumor volume was 1205.2 ± 581.3 mm^3^, 724.9 ± 337.6 mm^3^, 507.6 ± 367.0 mm^3^, and 245.3 ± 181.2 mm^3 ^in the control, "Songyou Yin," IFN-α, and combination groups, respectively. The combined therapy noticeably decreased the MMP2/TIMP2 ratio and prolonged the lifespan by 42.2%. Moreover, a significant (*P *< 0.001) reduction of microvessel density was found: 43.6 ± 8.5, 34.5 ± 5.9, 23.5 ± 5.6, and 18.2 ± 8.0 in the control and treatment groups, respectively.

**Conclusion:**

Palliative resection-stimulated HCC metastasis may occur, in part, by up-regulation of VEGF and MMP2/TIMP2. "Songyou Yin" reinforced the ability of IFN-α to inhibit the metastasis-enhancing potential induced by palliative resection, which indicated its potential postoperative use in patients with HCC.

## Background

Hepatocellular carcinoma (HCC) is the third most common cause of cancer-related deaths worldwide [1]. Liver resection is a widely accepted treatment modality for HCC; at the authors' institution, 53 patients with HCC with a 20-year survival all underwent curative resection [2]. However, the influence of liver resection on tumor growth and metastasis remains controversial. Increasing numbers of reports indicate that partial hepatectomy accelerates tumor growth and stimulates tumor metastasis [3-7], although one report claimed that major hepatic resection may suppress the growth of tumors remaining in the residual liver [8]. Another report indicated that hepatectomy prolongs survival of mice with induced liver metastases [9]. Finally, a report demonstrated that surgical therapy is associated with improved survival in patients with HCC [10].

Data from authors' institution (1958-2008, unpublished) revealed that the 5-year survival after palliative HCC resection (30.0%, *n *= 2754) was much lower than that following curative resection (52.6%, *n *= 5353). The clinical observation that patients with HCC receiving palliative resection (with grossly identified residual cancer) experienced dramatically increased metastases implies that palliative resection may enhance the metastatic potential of HCC, which is poorly understood to the best of our knowledge. Therefore, this study aimed to verify whether palliative resection enhances invasion and metastatic potential of residual HCC and to explore a novel approach for therapeutic intervention.

An orthotopic human HCC model in nude mice with high metastatic potential, which was established at the authors' institution, was used [11-13]. Our previous study showed that interferon-alfa-1b (IFN-α) [14] and the herbal extract "Songyou Yin [15]" (SYY) inhibit HCC growth, metastasis, and recurrence and prolong survival in the nude mice model system. Consequently, these two agents were used for this interventional study.

## Methods

### Animals

Male athymic BALB/c nu/nu mice, weighing 18-20 g at 5 weeks of age, were obtained from the Shanghai Institute of Materia Medica, Chinese Academy of Science. All mice were handled according to the recommendations of the National Institutes of Health Guidelines for Care and Use of Laboratory Animals. The experimental protocol was approved by the Shanghai Medical Experimental Animal Care Committee.

### HCC cell line and metastatic orthotopic tumor model in nude mice

At the authors' institution, a stepwise metastatic human HCC model system was established, which included a metastatic HCC model in nude mice LCI-D20 [11], an HCC cell line MHCC97 with high metastatic potential that originated from LCI-D20 tumor [12], and cell clone MHCC97H from its parent MHCC97, with a lung metastatic rate up to 100% using orthotopic inoculation [13]. Human HCC tumor models produced by MHCC97H were established in nude mice by orthotopic inoculation, as described previously [15]. The MHCC97H cells were maintained in Dulbecco modified Eagle medium (DMEM, Gibco-BRL, Gaithersburg, MD) without any antibiotic. HCC cell lines of HCCLM3 with higher invasiveness and HCC7721, HCC7402, and Hep3B with very low invasiveness were also prepared.

### Drugs

The Chinese herbal medicine formula "Songyou Yin" (SYY), a dietary component authorized by the Chinese State Food and Drug Administration (Grant No.G20070160), was originally designed to promote vital energy and to be considered as a nontoxic therapeutic supplement for cancer patients [15]. The mixture includes five Chinese medicinal herbal extracts, whose proportions (w/w) are as follows: *Salvia miltiorrhiza *Bge., 14.3%; *Astragalus membranaceus *Bge., 14.3%; *Lycium barbarum *L., 23.8%; *Crataegus pinnatifida *Bge., 23.8%; and *Trionyx sinensis *Wiegmann, 23.8% (all from China). The ethanol extract was prepared as follows: The dried and pulverized medicinal herbs were mixed together, and each batch was poached twice for a total of 3 h, then filtrated, concentrated, and soaked with ethanol for 12 h. Finally, SYY, with the level of relative density 1.36 (60°-80°C), was obtained and stored at 4°C before application in experiments. The SYY used in the in vitro and in vivo studies, with the same batch number (#060601), was produced by the Caitong Detang Chinese Traditional Medicine Pharmaceutical Factory, Shanghai, China. A 120-mg/mL sterilized SYY by two filtrations was prepared and then diluted cell culture medium or distilled water for further use. High-performance liquid chromatography (HPLC) fingerprinting of SYY and its five characteristic components was carried out by the Shanghai Institute of Materia Medica (SIMM), Chinese Academy of Sciences (CAS), China. The compound was diluted with distilled water for further use, which inhibits HCC cell lines MHCC97H with high invasive potential and Hep3B cells with very low invasiveness in vitro and inhibits HCC growth and metastasis in vivo [15]. Recombinant IFN-α (Sinogen, Kexing Bioproduct Company Ltd, Shenzhen, P.R. China) inhibits HCC, which may be attributable to anti-angiogenesis [14,16].

### Mice grouping and treatment

This study comprised 120 nude mice. In the first experiment, 48 nude mice bearing HCC xenografts were randomized into two groups 14 days after orthotopic implantation (24 mice/group), including a palliative resection group of mice undergoing partial HCC resection with preservation of 2 mm tumor [17] and a sham operation group (control) of mice having only exposure of the liver but no resection. In the second experiment, another 72 nude mice (14 days after orthotopic implantation) undergoing palliative resection were randomized into four groups according to the different therapies (18 mice/group). Therapy started on day 2 after palliative resection.

Control group mice each received 0.3 mL of distilled water via the oral gavage method once a day and were injected with sterile saline water (NS, 0.1 mL, subcutaneously) daily for 5 consecutive weeks 24 hours after resection.

Each mouse in the SYY group received 0.3 mL of "Songyou Yin" (3.6 g/kg/d/mouse) via the oral gavage method [15] and NS, 0.1 mL, subcutaneously daily.

The mice in the IFN-α group received 0.3 mL of distilled water via the oral gavage method and 0.1 mL of IFN-α (7.5 × 10^6 ^U/kg/d/mouse), subcutaneously daily [14].

Finally, the SYY + IFN-α group received the combined therapy with "Songyou Yin" and IFN-α as described for the groups given each agent alone.

### Parameters observed

In the first experiment, 6 mice of each group were humanely killed by cervical dislocation 35 days after palliative resection to detect pulmonary metastasis. The remaining mice (18/group) were kept alive and their lifespan was determined starting from the day of resection; the rate of prolonged life was calculated as done previously [15]. The in situ activities of membrane type 1-matrix metalloproteinase (MT1-MMP), matrix metalloproteinase 2 (MMP2), and protein levels of MMP2, tissue inhibitor of metalloproteinase 2 (TIMP2), vascular endothelial growth factor (VEGF), and endostatin in the grown tumors obtained from the implantation sites were determined.

In the second experiment, daily general observations and weekly body weights (BWs) of the mice were recorded; 6 mice from each group were humanely killed 48 hours after the final treatment. Tumor size was measured with calipers and volume was estimated by the formula *V *= L × H × W × 0.5236; the tumor inhibition rate was calculated [15]. The remaining 12 mice of each group were maintained on the designated therapies until death to determine their lifespan. Lung metastases, MMP2 activity, protein levels of MMP2, TIMP2, and VEGF, and microvessel density (MVD) were determined.

### Samples prepared

Serum, lung tissues, tumor tissues, and their protein extracts were harvested for in vitro and in vivo studies.

### In vitro invasion and migration assays

The cells of MHCC97H, HCCLM3, HCC7721, HCC7402, and Hep3B treated with serum from killed mice and cell culture medium supplemented with 10% human AB serum (control) for 72 hours were added to the upper chamber (100 μL DMEM, 5 × 10^4 ^cells/well) and 600 μL conditioned medium was added to the lower chamber. The invaded cells were fixed with methanol and stained with crystal violet solution after a 24-hour incubation. The migration assay was similar to the invasion assay, only without Matrigel. The results were expressed as the number of penetrated cells under microscope at ×200 magnification on five random fields and were presented as means ± SD of three assays [13].

### Hematoxylin and eosin stains

Paraffin blocks of 10% buffered formalin-fixed samples of tumor and lung tissues were prepared, serial sections were cut at 5 μm, and pulmonary metastatic nodules were verified with hematoxylin and eosin stain [18].

### MT1-MMP activity assay

The MT1-MMP activity assay kit (Amersham Biosciences, Buckinghamshire, UK) was used to detect active endogenous MT1-MMP in tissue extracts with analytical sensitivity of 0.7 ng/mL. The resultant color was read at 405 nm using an automated microplate reader (BioTek Instrument, Winooski, VT). The concentration of active MT1-MMP in the samples was determined by interpolation from a standard curve.

### Gelatin zymography assay

MMP2 activity in tumor tissue lysate was measured via Gelatin zymography assay [15]. The MMP activity was visualized as white proteolysis bands against the gel stained with Coomassie blue. The molecular weights of these bands indicating MMP2 activity were determined by molecular weight standards (Bio-Rad Laboratories, Hercules, CA). The relative MMP activity was quantitated by scanning the zymogram photograph on a gel documentation and analysis system (Alphalmager 2000, Alpha Innotech, San Leandro, CA). The area and optical density of each band was calculated and normalized by its own internal control; the digitized data of photographs presenting MMP2 activities in the control group were considered as 100%.

### Enzyme-linked immunosorbent assay

Protein levels of MMP2, TIMP2, VEGF, and endostatin in tumor extracts were measured via commercial available MMP2 (R&D Systems, Minneapolis, MN), TIMP2 (Amersham Biosciences, Buckinghamshire, UK), VEGF enzyme-linked immunosorbent assay (ELISA; R&D Systems, Minneapolis, MN), and endostatin (Chemicon International, Temecula, CA) kits. The mean of minimum detectable dose of MMP2, TIMP2, VEGF, and endostatin is 0.16 ng/mL, 3.2 ng/mL, 5 pg/mL, and 1.95 ng/mL, respectively. Absorbance was measured at 450 nm, using a microplate spectrophotometer with the correction wavelength of 570 nm. The assays were conducted in triplicate.

### MMP2 activity detected by ELISA

MMP2 activity was quantitated with a human MMP2 Activity ELISA System (Amersham Pharmacia Biotech, Piscataway, NJ) according to the manufacturer's instructions. The plate was read at 450 nm in a SPECTRAmax 250 Microplate Spectrophotometer (Molecular Devices, Sunnyvale, CA). The assays were conducted in triplicate.

### Immunohistochemistry assay

MVD (using CD34 immunostaining) was counted [19]. All slides were independently assessed by two board-certified pathologists who were blinded to the experiment. Any difference in the microvessel count was resolved by consensus.

### Statistical analysis

Data were analyzed by the statistical SAS software package (SAS 8.2) using ANOVAs, Student *t *test, and Kaplan-Meier method (log-rank test). All continuous variables were expressed as means ± SD or means ± SE. Statistical significance was set at *P *< 0.05.

## Results

### Palliative resection prolongs survival but increases lung metastases in nude mice with HCC

**Figure 1:**
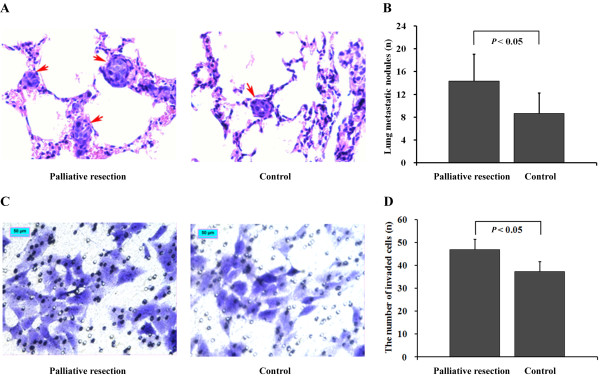
**Difference in tumor invasion and metastasis**. (A) The representative pulmonary metastatic nodules (*arrows*, original magnification, ×400). (B) The difference in lung metastasis (*P *< 0.05). (C) The penetrated cells (original magnification, ×200) treated by resection mice serum (left) was higher than that of control (right), bar, 50 μm. (D) The difference in invasiveness (*P *< 0.05). The bars indicate the means ± SD.

Increased pulmonary metastases were found in the palliative resection group (Figure 1A, left) as compared to the control (Figure 1A, right). The number of lung metastatic nodules was 14.3 ± 4.7 versus 8.7 ± 3.6 (*P *< 0.05, Figure 1B). However, the lifespan was longer in the palliative resection group compared with that in the control group (60.8 ± 2.7 d versus 51.3 ± 1.4 d, means ± SE, *P *< 0.05).

We found that cells (MHCC97H) treated with serum from the palliative resection group (Figure 1C, left) presented the most invasive potential through Matrigel as compared to cells in controls (Figure 1C, right), with invasive ability increased by 25.7% (46.9/37.3, 1.3-fold, *P *< 0.05, Figure 1D), which was intervalidated by several HCC cell lines (HCCLM3, HCC7721, HCC7402, and Hep3B). However, no significant differences were found in migration capacity of treated cells among groups.

### Palliative resection enhances MMP2 activity, elevates tumor protein levels of MMP2 and VEGF, and down-regulates TIMP2 in situ

**Figure 2:**
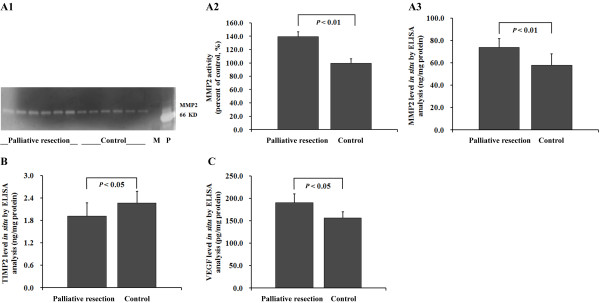
**Molecular alterations**. (A10 Gelatin zymography assay showed palliative resection activated MMP2. M, size marker; P, positive control. (A2) The percentage of MMP2 activity was elevated 1.4-fold as compared with control (*P *< 0.01). (A3) Palliative resection up-regulated the tumor MMP2 level (*P *< 0.01). (B) Palliative resection down-regulated tumor the TIMP2 level (*P *< 0.05). (C) Palliative resection up-regulated tumor the VEGF level (*P *< 0.05). The bars indicate the means ± SD.

Gelatin zymography assays clearly showed that tumor MMP2 activity was stronger in the palliative resection group (Figure 2A1); the normalized optical density of the band presenting MMP2 activity was significantly elevated (1.4-fold, *P *< 0.01, Figure 2A2), in accord with up-regulation of the MMP2 protein level in the palliative resection group, that is, 73.7 ± 8.0 ng/mg versus 57.9 ± 10.1 ng/mg (*P *< 0.01, Figure 2A3). TIMP2 was down-regulated with reductions in TIMP2 levels to 15.6% of control levels (*P *< 0.05, Figure 2B). Tumor VEGF levels were also up-regulated by palliative resection, being 185.7 ± 13.6 pg/mg versus 164.5 ± 12.5 pg/mg (*P *< 0.05, Figure 2C). However, the alteration of activated tumor MT1-MMP and endostatin levels did not reach statistical significance between the two groups.

### The combined therapy averts cancer-related BW loss and reinforces antitumor effects in nude mice model

**Figure 3:**
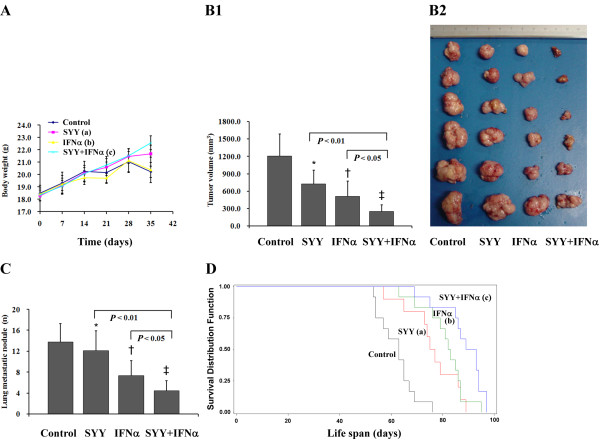
**Comparison of therapeutic effect**. (A) BW curves. The combined therapy noticeably minimized the BW loss. ^a^*P *< 0.01, ^b^*P *> 0.05, ^c^*P *< 0.001 compared with control at the end of 5 weeks of treatment. (B1) Tumor volumes. Tumor volumes in the control, SYY, IFN-α, and SYY + IFN-α groups were 1205.2 ± 581.3 mm^3^, 724.9 ± 337.6 mm^3^, 507.6 ± 367.0 mm^3^, and 245.3 ± 181.2 mm^3^, respectively. *P < 0.05, †*P *< 0.01, ‡*P *< 0.001 compared with control. (B2) Tumors in different groups with decreasing tendency. Tumor inhibition rate in 4 groups was 39.9% (SYY group), 57.9% (IFN-α group), and 79.6% (SYY + IFN-α group), respectively. (C) The mean number of lung metastatic nodules in 4 groups. Six mice from each group were humanely killed 48 hours after the final treatment. Paraffin blocks of 10% buffered formalin-fixed samples of lung tissues were prepared, serial sections were cut at 5 μm, and pulmonary metastatic nodules were verified with hematoxylin and eosin stain. *P > 0.05, †*P *< 0.05, ‡*P *< 0.01 compared with control. (D) Survival curves among groups. The remaining 12 mice of each group were maintained on the designated therapies until death to determine their lifespan. ^a^*P *< 0.05, ^b^*P *< 0.01, ^c^*P *< 0.001 compared with control. The bars indicate the means ± SD.

SYY inhibited the BW loss, IFN-α was not obviously related to the BW loss, but the combined therapy noticeably minimized the BW loss, with the maximum statistical difference of BW among groups observed at the fifth week after treatment (Figure 3A). Tumor volumes in the control, SYY, IFN-α, and SYY + IFN-α groups were 1205.2 ± 581.3 mm^3^, 724.9 ± 337.6 mm^3^, 507.6 ± 367.0 mm^3^, and 245.3 ± 181.2 mm^3^, respectively (*P *< 0.001, for the SYY + IFN-α group compared with control, Figure 3B1,2), with tumor inhibition rates of 39.9% (SYY group), 57.9% (IFN-α group), and 79.6% (SYY + IFN-α group). At the end of the fifth week, the mean number of lung metastatic nodules was reduced by 12.3%, 46.4%, and 67.4%, respectively, compared to the control (Figure 3C). The lifespan was significantly extended in treatment groups (Figure 3D), and the rate of prolonged life was 42.2% in the SYY + IFN-α group.

### Inhibitory effect of therapies correlates to the inhibition of MMP2 and VEGF

**Figure 4:**
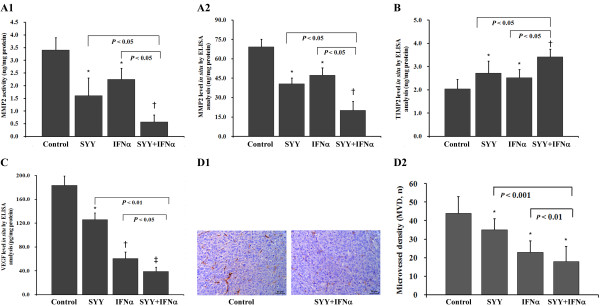
**Detection of VEGF, MMP2, TIMP2, and MVD**. (A1) Tumor MMP2 activities. *P < 0.05, †*P *< 0.01 compared with control. (A2) Tumor MMP2 levels. *P < 0.05, †*P *< 0.01 compared with control. (B) Tumor TIMP2 levels. *P < 0.01, †*P *< 0.001 compared with control. (C) Tumor VEGF levels. *P < 0.05, †*P *< 0.01, ‡*P *< 0.001 compared with control. (D1) Representative CD34 immunostained intratumoral microvessels (original magnification, ×200) in control group (left) and in the SYY + IFN-α group (right), bar, 50 μm. (D2) Tumor microvessel density (MVD). *P < 0.001 compared with control. The bars indicate the means ± SD.

Our data revealed that both tumor MMP2 activity (Figure 4A1) and MMP2 (Figure 4A2) and VEGF (Figure 4C) protein levels were significantly down-regulated in treatment groups, whereas TIMP2 (Figure 4B) levels were up-regulated when compared to the controls. According to the inhibitory effects of SYY and IFN-α, we found that SYY was more effective on MMP2 (Figure 4A1,2), IFN-α was more effective on VEGF (Figure 4C), and the combined therapy was more effective than any agent alone. On the other hand, tumor MVD (Figure 4D1) decreased in the treated groups, being 34.5 ± 5.9, 23.5 ± 5.6, and 18.2 ± 8.0, respectively, as compared to control (43.6 ± 8.5, *P *< 0.001, Figure 4D2).

No animal experienced therapy-related side effects, BW loss of > 10%, anemia, neutropenia, thrombocytopenia, or abnormal serum transaminases after the treatment regimen in this study (data not shown).

## Discussion

Hepatectomy is a standard treatment for patients with HCC [2,10]. However, even in patients undergoing curative resection, the procedure is only potentially curative due to the existence of tumor cells or clinically undetectable residual intrahepatic lesions [2,3]. Our clinical observation showed that after palliative resection the patients experienced dramatically increased metastases without systemically effective treatments from the oncologic standpoint. However, several studies have demonstrated survival benefits from palliative liver resection [20-23]. Thus, the effectiveness of palliative resection for HCC remains controversial. This study was designed to evaluate the progression of residual tumor after palliative resection and to explore an interventional approach in a high metastatic human HCC model system that has been successfully used for studies of HCC-related invasion, metastasis [23], and screening of therapeutic agents for HCC [14,15,18,24].

Palliative resection has been applied to treat HCC and produced marked survival benefits when combined with adjunct IFN-α/5-fluorouracil therapy [21,22]. It has been postulated that the survival prolongation after palliative resection may be partially due to tumor-debulking surgery [21]. In this study, therefore, we investigated the benefit from palliative resection in nude mice bearing human HCC and found the lifespan was 60.8 ± 2.7 days in palliative resection compared with 51.3 ± 1.4 days in controls. Our result was consistent with previous reports [21,22], which implied that patients with HCC could benefit from palliative resection. In contrast, some studies have reported that partial hepatectomy accelerates tumor growth and stimulates tumor metastasis [3-7]. Our experimental research revealed that palliative resection is followed by acceleration of metastatic processes in the lungs. The increased pulmonary metastatic nodules were found in the palliative resection group (14.3 ± 4.7) compared to controls (8.7 ± 3.6), which may be partially due to breakdown of tumor MMP2/TIMP2 balance and in situ up-regulation of VEGF that was consistent with a recent report that confirmed increased VEGF transcription in residual HCC after hepatectomy in a small animal model [25].

The higher ratio of MMP/TIMP has been related to a poorer prognosis in HCC [26]. Our data revealed that palliative resection activated MMP2 and down-regulated TIMP2. Lower TIMP2 levels might cause MMP2 activation, which resulted in degradation of the extracellular matrix and increased angiogenesis. VEGF, a potent stimulator of tumor angiogenesis, is believed to have a major role in HCC angiogenesis, growth, and metastasis. In this study, we found palliative liver resection elevated tumor VEGF levels markedly, in accordance with the literature [25]. The alteration of endostatin (a potent angiogenesis inhibitor) and MT1-MMP (an activator of MMP2) was minor, which indicated that elevated tumor VEGF and increased MMP2 activity were not directly due to endostatin and MT1-MMP, whose detailed molecular mechanism remains to be elucidated.

In most studies, palliative resection for HCC was combined with chemotherapy or biotherapy, such as IFN-α [21,22]. Our previous studies revealed that IFN-α inhibited HCC and extended the lifespan of mice and could be attributed to anti-angiogenesis [14,16]; SYY directly down-regulated MMP2 and VEGF and inhibited HCC growth and metastasis [15]. Others also reported that herbal medicine could inhibit the growth of MHCC97H cells used in this study [27] and could inhibit the invasiveness potential of HCC cells via MMP2 inhibition [28]. Based on our in vitro and in vivo studies, we considered that inhibition of the metastasis-enhancing effects due to dysregulation of MMP2/TIMP2 and VEGF might further improve operative efficacy in patients with HCC. Fortunately, the study showed that combined therapy with SYY plus IFN-α was more effective than any single agent on residual HCC following palliative resection. This inhibitory effect of combined therapy on progression of preexisting HCC was partially associated with a decreased VEGF level and lower MVD, which is in keeping with recent experimental evidence that growth of small nests of cells can be stunted by a variety of agents that have the common ability to inhibit angiogenesis [29]. Moreover, in our study, the side effects of therapy in mice were well tolerated [14,15]. The weight changes in mice observed in this research showed that SSY and the combined therapy noticeably minimized BW loss at the fifth week after treatment, which suggested that we should administer herbal medicine for a long period. Consequently, more attention should be paid to the active role of Chinese herbal medications in combination therapy for patients with HCC [30].

We are also aware of some limitations in our study. First, we could not sufficiently elucidate the exact mechanism of the metastasis-enhancing potential of residual tumor. Further basic research for the stimulating effect is needed. Second, we did not offer a detailed explanation for how SYY reinforced IFN-α to inhibit HCC. Third, because the study used nude mice the ability to provide robust evidence is limited. Therefore, large-scale, multicenter, placebo-controlled, and prospective studies are needed to test our results. Despite these limitations, we believe that the current study provides preliminary and powerful data to support future evaluation of SYY in combination with IFN-α for HCC in a large cohort, randomized clinical trial that has been performed in Zhongshan Hospital of Fudan University, Shanghai, PR China.

In summary, palliative resection induced the metastasis-enhancing potential of residual HCC via breakdown of the MMP2/TIMP2 balance and up-regulation of VEGF, but the precise mechanism of this stimulating effect remains to be elucidated. Combination therapy with SYY plus IFN-α, by regulating the MMP2/TIMP2 ratio and VEGF expression, could be an effective therapeutic strategy to reverse the tumor-enhancing effect derived from hepatectomy. The results indicate the potential use of this therapy to improve the postoperative prognosis of patients with HCC.

## Conclusions

This study showed that palliative resection-accelerated HCC metastasis may be attributed, in part, to up-regulation of VEGF and MMP2/TIMP2; SYY reinforced IFN-α to inhibit the metastasis-enhancing potential. Therefore, the combined therapy of SYY plus IFN-α may be used after hepatectomy for patients with HCC.

## Abbreviations

HCC: hepatocellular carcinoma; IFN-α: interferon-alfa-1b; MMP2: matrix metalloproteinase 2; TIMP2: tissue inhibitor of metalloproteinase 2; MT1-MMP: membrane type 1-matrix metalloproteinase; VEGF: vascular endothelial growth factor; MVD: microvessel density; DMEM: Dulbecco modified Eagle medium

## Competing interests

The authors declare that they have no competing interests.

## Authors' contributions

QZ and ZYT organized the study, planned the experiments, performed the statistical analysis and helped to write the manuscript. XYH, ZLH, and LW contributed to the design of this study, selected the samples, performed the statistical analysis, and drafted the manuscript. YHX participated in the design and coordination of the study. XYH and KXA contributed to the interpretation of the immunohistochemical data and helped write the manuscript. All authors read and approved the final manuscript.

## Pre-publication history

The pre-publication history for this paper can be accessed here:

http://www.biomedcentral.com/1471-2407/10/580/prepub

## References

[B1] Parkin DM Bray F Ferlay J Pisani P Global cancer statistics, 2002 CA Cancer J Clin 2005 55 74 108 10.3322/canjclin.55.2.74 15761078

[B2] Zhou XD Tang ZY Ma ZC Fan J Wu ZQ Qin LX Zhou J Yu Y Sun HC Qiu SJ Twenty-year survivors after resection for hepatocellular carcinoma-analysis of 53 cases J Cancer Res Clin Oncol 2009 135 1067 1072 10.1007/s00432-009-0546-z 19294419 PMC12160204

[B3] García-Alonso I Palomares T Alonso A Echenique-Elizondo M Caramés J Castro B Méndez J Effect of liver resection on the progression and growth of rhabdomyosarcoma metastases in a rat model J Surg Res 2008 148 185 190 10.1016/j.jss.2007.06.026 18028954

[B4] de Jong KP Lont HE Bijma AM Brouwers MA de Vries EG van Veen ML Marquet RL Slooff MJ Terpstra OT The effect of partial hepatectomy on tumor growth in rats: *in vivo *and *in vitro *studies Hepatology 1995 22 4 Pt 1 1263 1272 10.1002/hep.1840220436 7557880

[B5] Harun N Nikfarjam M Muralidharan V Christophi C Liver regeneration stimulates tumor metastases J Surg Res 2007 1 38 284 290 10.1016/j.jss.2006.06.024 17254608

[B6] Picardo A Karpoff HM Ng B Lee J Brennan MF Fong Y Partial hepatectomy accelerates local tumor growth: Potential roles of local cytokine activation Surgery 1998 124 57 64 9663252

[B7] García-Alonso I Palomares T Alonso A Portugal V Castro B Caramés J Méndez J Effect of hepatic resection on development of liver metastasis Rev Esp Enferm Dig 2003 95 765 776 14640874

[B8] Yokoyama S Goto CL Chen TL Pan TL Kawano K Kitano S Major hepatic resection may suppress the growth of tumors remaining in the residual liver Br J Cancer 2000 83 1096 1101 10.1054/bjoc.2000.1379 10993659 PMC2363560

[B9] Castillo MH Doerr RJ Paolini N Jr Cohens S Goldrosen M Hepatectomy prolongs survival of mice with induced liver metastases Arch Surg 1989 124 167 169 2916937 10.1001/archsurg.1989.01410020037005

[B10] Liu JH Chen PW Asch SM Busuttil RW Ko CY Surgery for hepatocellular carcinoma: does it improve survival? Ann Surg Oncol 2004 11 298 303 10.1245/ASO.2004.03.042 14993025

[B11] Sun FX Tang ZY Liu KD Ye SL Xue Q Gao DM Ma ZC Establishment of a metastatic model of human hepatocellular carcinoma in nude mice via orthotopic implantation of histologically intact tissues Int J Cancer 1996 66 239 243 10.1002/(SICI)1097-0215(19960410)66:2<239::AID-IJC17>3.0.CO;2-7 8603818

[B12] Tian J Tang ZY Ye SL Liu YK Lin ZY Chen J Xue Q New human hepatocellular carcinoma (HCC) cell line with highly metastatic potential (MHCC97) and its expressions of the factors associated with metastasis Br J Cancer 1999 81 814 821 10.1038/sj.bjc.6690769 10555751 PMC2374300

[B13] Li Y Tang ZY Ye SL Liu YK Chen J Xue Q Chen J Gao DM Bao WH Establishment of cell clones with different metastatic potential from the metastatic hepatocellular carcinoma cell line MHCC97 World J Gastroenterol 2001 7 630 636 11819844 10.3748/wjg.v7.i5.630 PMC4695564

[B14] Wang L Tang ZY Qin LX Wu XF Sun HC Xue Q Ye SL High-dose and long-term therapy with interferon-alfa inhibits tumor growth and recurrence in nude mice bearing human hepatocellular carcinoma xenografts with high metastatic potential Hepatology 2000 32 43 48 10.1053/jhep.2000.8525 10869287

[B15] Huang XY Wang L Huang ZL Zheng Q Li QS Tang ZY Herbal extract "Songyou Yin" inhibits tumor growth and prolongs survival in nude mice bearing human hepatocellular carcinoma xenograft with high metastatic potential J Cancer Res Clin Oncol 2009 135 1245 1255 10.1007/s00432-009-0566-8 19277711 PMC12160221

[B16] Wang L Wu WZ Sun HC Wu XF Qin LX Liu YK Liu KD Tang ZY Mechanism of interferon alpha on inhibition of metastasis and angiogenesis of hepatocellular carcinoma after curative resection in nude mice J Gastrointest Surg 2003 7 587 594 10.1016/S1091-255X(03)00072-6 12850669

[B17] Broomfield S Currie A van der Most RG Brown M van Bruggen I Robinson BW Lake RA Partial, but not complete, tumor-debulking surgery promotes protective antitumor memory when combined with chemotherapy and adjuvant immunotherapy Cancer Res 2005 65 7580 7584 16140921 10.1158/0008-5472.CAN-05-0328

[B18] Zhou J Tang ZY Fan J Wu ZQ Ji Y Xiao YS Shi YH Li XM Sun QM Liu YK Capecitabine inhibits postoperative recurrence and metastasis after liver cancer resection in nude mice with relation to the expression of platelet-derived endothelial cell growth factor Clin Cancer Res 2003 9 16 Pt 1 6030 6037 14676129

[B19] Weidner N Semple JP Welch WR Folkman J Tumor angiogenesis and metastasis--correlation in invasive breast carcinoma N Engl J Med 1991 324 1 8 10.1056/NEJM199101033240101 1701519

[B20] Gotohda N Kinoshita T Konishi M Nakagohri T Takahashi S Furuse J Ishii H Yoshino M New indication for reduction surgery in patients with advanced hepatocellular carcinoma with major vascular involvement World J Surg 2006 30 431 438 10.1007/s00268-005-0250-3 16479350

[B21] Nagano H Miyamoto A Wada H Ota H Marubashi S Takeda Y Dono K Umeshita K Sakon M Monden M Interferon-alpha and 5-fluorouracil combination therapy after palliative hepatic resection in patients with advanced hepatocellular carcinoma, portal venous tumor thrombus in the major trunk, and multiple nodules Cancer 2007 110 2493 2501 10.1002/cncr.23033 17941012

[B22] Nagano H Sakon M Eguchi H Kondo M Yamamoto T Ota H Nakamura M Wada H Damdinsuren B Marubashi S Hepatic resection followed by IFN-alpha and 5-FU for advanced hepatocellular carcinoma with tumor thrombus in the major portal branch Hepatogastroenterology 2007 54 172 179 17419255

[B23] Ye QH Qin LX Forgues M He P Kim JW Peng AC Simon R Li Y Robles AI Chen Y Predicting hepatitis B virus-positive metastatic hepatocellular carcinomas using gene expression profiling and supervised machine learning Nat Med 2003 9 416 423 10.1038/nm843 12640447

[B24] Wang Z Zhou J Fan J Qiu SJ Yu Y Huang XW Tang ZY Effect of rapamycin alone and in combination with sorafenib in an orthotopic model of human hepatocellular carcinoma Clin Cancer Res 2008 14 5124 5130 10.1158/1078-0432.CCR-07-4774 18698030

[B25] Perry KA Enestvedt CK Hosack LW Pham TH Diggs BS Teh S Orloff S Winn S Hunter JG Sheppard BC Increased vascular endothelial growth factor transcription in residual hepatocellular carcinoma after open versus laparoscopic hepatectomy in a small animal model Surg Endosc 2010 24 1151 1157 10.1007/s00464-009-0742-6 19915910

[B26] Altadill A Rodríguez M González LO Junquera S Corte MD González-Dieguez ML Linares A Barbón E Fresno-Forcelledo M Rodrigo L Liver expression of matrix metalloproteases and their inhibitors in hepatocellular carcinoma Dig Liver Dis 2009 41 740 748 10.1016/j.dld.2009.01.016 19372066

[B27] Yuan A Li Z Li X Yi S Wang S Shi K Bian J Distinct effect of Chrysanthemum indicum Linné extracts on isoproterenol-induced growth of human hepatocellular carcinoma cells Oncol Rep 2009 22 1357 1363 10.3892/or_00000521 19885587

[B28] Ha KT Kim JK Kang SK Kim DW Lee YC Kim HM Kim CH Inhibitory effect of Sihoga-Yonggol-Moryo-Tang on matrix metalloproteinase-2 and -9 activities and invasiveness potential of hepatocellular carcinoma Pharmacol Res 2004 50 279 285 10.1016/j.phrs.2004.02.006 15225671

[B29] Xu Y Wen Z Xu Z Chitosan nanoparticles inhibit the growth of human hepatocellular carcinoma xenografts through an antiangiogenic mechanism Anticancer Res 2009 29 5103 5109 20044623

[B30] Yu Y Lang Q Chen Z Li B Yu C Zhu D Zhai X Ling C The efficacy for unresectable hepatocellular carcinoma may be improved by transcatheter arterial chemoembolization in combination with a traditional Chinese herbal medicine formula Cancer 2009 115 5132 5138 10.1002/cncr.24567 19672999

